# Interactions of Neuropathogenic *Escherichia coli* K1 (RS218) and Its Derivatives Lacking Genomic Islands with Phagocytic *Acanthamoeba castellanii* and Nonphagocytic Brain Endothelial Cells

**DOI:** 10.1155/2014/265424

**Published:** 2014-04-10

**Authors:** Farzana Abubakar Yousuf, Zuhair Yousuf, Junaid Iqbal, Ruqaiyyah Siddiqui, Hafsa Khan, Naveed Ahmed Khan

**Affiliations:** Department of Biological and Biomedical Sciences, Aga Khan University, Stadium Road, Karachi 74800, Pakistan

## Abstract

Here we determined the role of various genomic islands in *E. coli* K1 interactions with phagocytic *A. castellanii* and nonphagocytic brain microvascular endothelial cells. The findings revealed that the genomic islands deletion mutants of RS218 related to toxins (peptide toxin, **α**-hemolysin), adhesins (P fimbriae, F17-like fimbriae, nonfimbrial adhesins, Hek, and hemagglutinin), protein secretion system (T1SS for hemolysin), invasins (IbeA, CNF1), metabolism (D-serine catabolism, dihydroxyacetone, glycerol, and glyoxylate metabolism) showed reduced interactions with both *A. castellanii* and brain microvascular endothelial cells. Interestingly, the deletion of RS218-derived genomic island 21 containing adhesins (P fimbriae, F17-like fimbriae, nonfimbrial adhesins, Hek, and hemagglutinin), protein secretion system (T1SS for hemolysin), invasins (CNF1), metabolism (D-serine catabolism) abolished *E. coli* K1-mediated HBMEC cytotoxicity in a CNF1-independent manner. Therefore, the characterization of these genomic islands should reveal mechanisms of evolutionary gain for *E. coli* K1 pathogenicity.

## 1. Introduction

The incidence of bacterial meningitis has increased in recent years, despite improvement in the antimicrobial chemotherapy.* Escherichia coli* is the second leading cause of bacterial meningitis, behind group B streptococcus (GBS), and contributes significantly to morbidity and mortality [1–5]. The pathogenesis of* E. coli* meningitis involves a series of steps: (i) bacterial entry of the intestinal tract, following accumulation from the mother's flora during delivery or from the environment, but this may also occur via the urinary tract (20% of cases), or* in utero* following maternal bacteraemia, (ii) bacterial colonization of the mucosa and invasion of the intravascular space, (iii) survival and multiplication in the bloodstream resulting in bacteraemia, (iv) bacterial crossing of the blood-brain barrier and invasion of the central nervous system resulting in inflammation, pleocytosis, and neuronal injury that ultimately leads to death; however, there are a few survivors with neurological sequelae [2, 6–9]. Each of the aforementioned steps is highly complex that requires specific pathogen-host interactions involving a plethora of molecules associated with different stages of meningitis development. For example,* E. coli* crossing of the brain microvascular endothelial cells (BMEC) that constitute the blood-brain barrier can be divided into four steps: (i)* E. coli* binding to the BMEC using a variety of adhesins, (ii)* E. coli* invading the BMEC using a variety of invasins, (iii)* E. coli*-containing vacuole intracellular trafficking across the cell, that is, from the apical side to the basolateral side, and (iv)* E. coli* exiting on the central nervous system side [7]. Evolutionarily, it is not clear how* E. coli* has developed such a sophisticated mechanism(s) resulting in virulence to produce human and animal infection. In this regard, amoebae have been suggested as the “training ground” during evolution of intracellular bacterial pathogens [10]. Thus, it is hypothesized that the fragility of non-spore-forming bacteria to survive hostile conditions and inability to disperse to favorable environments may have led to their evolutionary need to associate with a hardy host and/or a biological reservoir such as* Acanthamoeba* to remain viable intracellularly in the face of harsh conditions, whereas bacteria use* Acanthamoeba* to enhance their pathogenicity. In such harsh environmental conditions, it is plausible that* Acanthamoeba* harbor bacteria and transmit them to the susceptible hosts. Once in the gut, amoebae may lyse resulting in bacterial colonization. Thus it is important to understand interactions of pathogenic bacteria with* Acanthamoeba* that may have influenced evolutionary gain of* E. coli* K1 pathogenicity and their potential role in the transmission.

Research over the last few decades has identified bacterial virulence determinants that are associated with* E. coli* K1 meningitis including fimbrial protein, FimH, outer membrane protein A (OmpA), cytotoxic necrotizing factor 1 (Cnf-1), Ibe proteins, AslA and TraJ, and the K1 capsular polysaccharide [7, 11]. The* E. coli* K1 genome has been sequenced (http://www.genome.wisc.edu) and 22 genomic islands consisting of ~793 kb have been identified that are present in meningitis-causing* E. coli* RS218 but absent in the nonpathogenic* E. coli* MG1655 [12]. Thus it is reasonable to suspect the presence of several additional virulence determinants involved in neuropathogenic* E. coli* K1 pathogenesis. In the present study, we determined the role of various genomic islands in* E. coli* K1 interactions with an environmental phagocyte,* Acanthamoeba castellanii,* and nonphagocytic brain endothelial cells.

## 2. Methods 

A clinical isolate of* A. castellanii* belonging to T4 genotype, originally isolated from a keratitis patient (American Type Culture Collection, ATCC 50492) was used in the present study.* A. castellanii* was cultured in the PYG medium containing (0.75% (w/v) proteose peptone, 0.75% (w/v) yeast extract and 1.5% (w/v) glucose)) [13].

**Figure 1:**
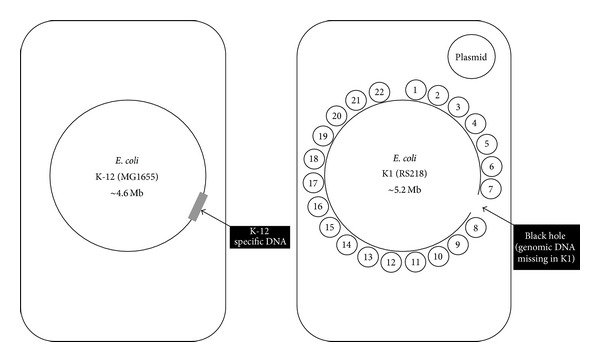
Comparative genomes of* E. coli *K1 and* E. coli *K-12. Common DNA: large circle represents the common* E. coli *backbone. K1-specific elements: 22 (numbered) islands of K1-specific DNA are represented as smaller circles and a plasmid. K-12-specific elements: 20 kb K-12 specific DNA is indicated in grey (referred to as a “black hole” in K1 genome). Adopted from Xie et al. [12].

*E. coli* K1 strain E44, used in the present study, is a rifampicin-resistant mutant of strain RS218 (serotype O18:K1:H7). This strain is a clinical isolate from the cerebrospinal fluid of a neonate with meningitis. A series of seventeen strains of* E. coli* RS218-derived genomic islands (RDIs), using a comparative genome analysis of meningitis-causing* E. coli* K1 strain RS218 (O18:K1:H7), together with a plasmid-cured* E. coli* K1 strain RS218 were obtained from the laboratory of Kwang S. Kim (Johns Hopkins University) as previously described [12, 14, 15] (Figure 1 and Table 1). To this end, a 20 kb K-12 specific DNA (genomic deletion in K1) was inserted into K1 genome using P1 transduction. Briefly, island boundaries were identified using genetic mapping, followed by insertion of antibiotic tag in the corresponding region of either K1 strain E44 or K-12 strain MG1655 using *λ*Red recombinase. P1 *ϕ* transduction from MG1655 to* E. coli *K1 results in the deletion of K1-specific genes, replaced with K-12 genes (Figure 1 and Table 1) as previously described [12, 14]. All bacterial strains were aerobically grown for approximately 14 h in Luria-Bertani (LB) broth at 37°C.

Primary brain microvascular endothelial cells (BMEC) were isolated from seizure patient who had undergone cerebral cortex resection as previously described [16]. The BMECs were grown in T-75 tissue culture flasks in RPMI-1640 containing 10% heat inactivated fetal bovine serum, 10% Nu-serum, 2 mM glutamine, 1 mM Na-pyruvate, 100 U/mL penicillin, 100 *μ*g/mL streptomycin, nonessential amino acids, and vitamins as previously described [16, 17].

For cytotoxicity assays, 1 × 10^5^ BMECs per well per 0.5 mL were cultured in 24-well plates and incubated at 37°C in a 5% CO_2_. Under these conditions, BMECs formed complete monolayers within 48 h.* E. coli* K1 and its derivative mutants were grown in LB for overnight and the optical density was adjusted to 0.22 absorbance at wavelength of 595 nm (equivalent to approximately 10^8^ colony forming units (c.f.u.) per mL) [15]. Next, 10 *μ*L containing 10^6^ c.f.u. were transferred to 490 *μ*L RPMI 1640, supplemented with 10% heat-inactivated fetal bovine serum, and inoculated in each well of a 24-well plate containing BMEC monolayers. Plates were incubated at 37°C in a 5% CO_2_ incubator and monitored for monolayer disruptions over the period of 24 h. Next, the supernatants were collected and centrifuged at 12000 ×g for 5 min to remove cellular debris. Cytotoxic effects were determined by estimating the release of cytosolic lactate dehydrogenase release in the medium (Cytotoxicity Detection kit; Roche Applied Sciences). The percent cytotoxicity was calculated as follows: % cytotoxicity = (sample value−control value)/(total LDH release−control value) × 100. Control values were obtained by incubating BMEC monolayers with 500 *μ*L RPMI-1640 alone and total LDH was released from BMEC by completely lysing them in 500 *μ*L 1% Triton X-100.

Assays were performed to determine* E. coli* K1 and its derivative mutant association with* A. castellanii* and human BMEC. For* A. castellanii*, amoebae were maintained in the trophozoite stage in tissue culture flasks in the PYG medium. Upon confluency, the unbound amoebae were aspirated and growing trophozoites were rinsed with phosphate buffer saline (PBS) pH 7.4. Next, 5 mL of PYG medium was added to the flask and trophozoites were chilled on ice for 20 min and pelleted by centrifugation at 900 ×g for 5 min. The cell pellet was resuspended in 1 mL of PBS and the number of amoebae was counted using a haemocytometer.* E. coli* K1 and its derivative mutants (10^7^ c.f.u.) were incubated with* A. castellanii *(10^6^ cells) at 30°C for 1 h. Following 1 h of incubation, amoebae were centrifuged at 2000 ×g for 5 min. The supernatants were aspirated and pellet resuspended in 0.5 mL of PBS. This process was repeated 3× to remove nonadherent bacteria. The amoebae were counted using a haemocytometer and then lysed by adding SDS (0.5% final concentration) for 10 min at room temperature, which does not affect* E. coli* viability [18]. The lysates containing bacteria were plated on nutrient agar plates and colonies enumerated the next day [18]. The bacterial colony forming units associated with* A. castellanii* were calculated as follows: (number of bacterial c.f.u./number of amoebae) × 100 = bacterial association of* A. castellanii* (percentage). To study* E. coli *interactions with human cells, BMECs were grown to confluent monolayers in 24-well plates. Next,* E. coli* K1 and its derivative mutants were incubated with human BMEC (10^7^ c.f.u. per well in 0.5 mL) and plates were incubated at 37°C in 5% CO_2_ incubator. After 60 min of incubation, the monolayers were washed with PBS and BMECs were lysed by adding 500 *μ*L of distilled water for 30 min together with gentle scraping of the well. The lysate was plated on nutrient agar and the colonies were counted the next day. The bacterial colony forming units associated with BMEC for the wild type* E. coli* K1 was arbitrarily set at 100% and the results of the mutants strains are expressed as the relative change.

Assays were performed to determine* E. coli* K1 and its derivative mutant uptake/invasion by* A. castellanii* and human BMEC. Briefly, amoebae were incubated with* E. coli* and its derivative mutants as described for association assays. After washing for 3×, the extracellular bacteria were killed by adding gentamicin (100 *μ*g per mL in PBS for 60 min. Finally, amoebae and bacteria were enumerated as described above. The bacterial colony forming units invading* A. castellanii* were calculated as follows: (number of bacterial c.f.u./number of amoebae) × 100 = bacterial invasion of* A. castellanii*. For human BMEC,* E. coli* K1 and its derivative mutants were incubated with BMEC. After 60 min of incubation, the monolayers were washed with PBS and incubated with gentamicin (100 *μ*g per mL in RPMI-1640) for 60 min to kill extracellular bacteria. The wells were then washed twice with RPMI-1640 and bacterial colony forming units were determined as described above.

Assays were performed to determine* E. coli* K1 and its derivative mutant intracellular survival of* A. castellanii* and human BMEC. Briefly, following invasion assays,* A. castellanii *was washed 3× with PBS and incubated in 0.5 mL of PBS for 24 h at 30°C. Finally, amoebae and bacteria were enumerated. The bacterial colony forming units surviving inside* A. castellanii* were calculated as follows: (number of bacterial c.f.u./number of amoebae) × 100 = bacterial survival of* A. castellanii*. For human BMEC, following invasion assay, the monolayers were washed twice with RPMI-1640 and BMECs were incubated in 0.5 mL of RPMI-1640 for 4 h at 37°C in a 5% CO_2_ incubator. Finally, bacterial colony forming units were determined.

Encystment assays were performed to evaluate the ability of* E. coli* K1 and its derivative mutants to survive inside* A. castellanii *cysts. In brief, following invasion assays, the mixtures were transferred onto nonnutrient agar plates (prepared using 3% (w/v) purified agar). The plates were incubated at room temperature for up to 10 days. This allowed a complete encystment of* A. castellanii *trophozoites into the cyst form, as observed visually under a phase-contrast microscope. Cysts were then gently scraped off the agar surface using a cell scraper by adding 5 mL of dH_2_O and collected by centrifugation at 2000 ×g for 10 min and resuspended in 0.5 mL of dH_2_O and counted using a haemocytometer. The cysts were treated with SDS (0.5% final concentration) and the bacterial colony forming units were determined by plating on nutrient agar plates. The bacterial colony forming units surviving intracellular of* A. castellanii* cysts were calculated as follows: (number of bacterial c.f.u./number of amoebae cysts) × 100 = bacterial survival of* A. castellanii* cysts. Statistical significance for differences was evaluated using Student's* t*-test in Excel. A critical value of *P* < 0.05 was used for all analysis using paired* t*-test, one-tailed distribution. Data are presented as the mean ± standard error.

## 3. Results 

Plasmids, bacteriophages, and pathogenicity islands are genomic additions that contribute to the evolution of bacterial pathogens. In addition to acquired virulence genes on a plasmid, chromosomal genes are also lost via deletions. The formation of these “black holes,” that is, deletions of genes that are detrimental to a pathogenic lifestyle, provides an evolutionary pathway that enables a pathogen to enhance virulence [14]. For* E. coli* interactions with* A. castellanii*, the findings revealed that the deletion of RDIs 2, 9, 10, 15, and 17 resulted in significantly reduced association, while RDI 21 exhibited increased association with* A. castellanii* (*P* < 0.05 using paired* t*-test, one-tailed distribution) (Table 2). For invasion of* A. castellanii*, plasmid cured RS218, the wild type* E. coli* K1 in which black hole (region of genomic deletion in K1 but present in K-12) was filled with K-12 HB101 DNA, deletions of RDIs 2, 9, 10, 12, 15, 18, 21, and 22 caused significantly reduced invasion of* A. castellanii* compared with the wild type* E. coli* K1 (*P* < 0.05) (Table 2), while the deletion of RDI 4 resulted in increased invasion. In survival assays, plasmid cured RS218, deletions of RDIs 9, 10, 18, 21, and 22 lead to significantly reduced survival of* A. castellanii* compared with the wild type* E. coli* K1 (*P* < 0.05) (Table 2), while* A. castellanii* remained intact and viable after survival prior to lysing with the SDS. When tested for their ability to survive transformation of* A. castellanii *trophozoite into cyst forms, the findings revealed that plasmid cured RS218, deletions of RDIs 2, 4, 9, 10, 16, 18, 21, and 22 caused significantly reduced recovery from* A. castellanii* cysts compared with the wild type* E. coli* K1 (*P* < 0.05) (Table 2). In contrast, the wild type* E. coli* K1 in which black hole was filled with K-12 HB101 DNA and the deletion of RDI 17 showed increased recovery from* A. castellanii* cysts compared with the wild type* E. coli* K1 (*P* < 0.05) (Table 2).

For* E. coli* interactions with BMEC, the plasmid cured RS218, the wild type* E. coli* K1 in which black hole was filled with K-12 HB101 DNA and deletions of RDIs 10, 12, 15, 16, 21, and 22 resulted in significantly reduced association with BMEC (*P* < 0.05) (Table 3). For invasion of BMEC, plasmid cured RS218, the wild type* E. coli* K1 in which black hole was filled with K-12 HB101 DNA and deletions of RDIs 2, 10, 17, 21, and 22 lead to significantly reduced invasion of BMEC compared with the wild type* E. coli* K1 (*P* < 0.05) (Table 2), while the deletion of RDI 18 showed increased invasion. In survival assays, plasmid cured RS218, deletions of RDIs 10, 17, 21, and 22 caused significantly reduced survival inside BMEC compared with the wild type* E. coli* K1 (*P* < 0.05) (Table 2). In contrast, the wild type* E. coli* K1 in which black hole was filled with K-12 HB101 DNA and the deletion of RDI 16 showed increased recovery from* A. castellanii* cysts compared with the wild type* E. coli* K1 (*P* < 0.05) (Table 2).

**Figure 2:**
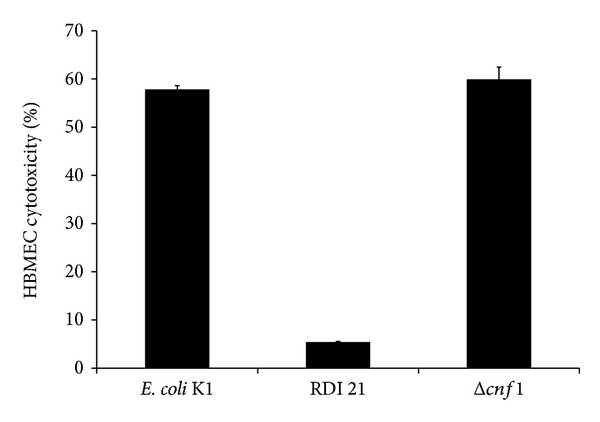
*Escherichia coli* K1-mediated human brain microvascular endothelial cell cytotoxicity was abolished with the deletion of RS218-derived genomic island 21.* E. coli *K1 strain E44 (10^6^ c.f.u.) was added to confluent cultures of primary human brain microvascular endothelial cells (HBMEC) grown in 24-well plates. Plates were incubated in a CO_2_ incubator for 24 h. At the end of the incubation, supernatants were tested for LDH release and converted to percentage cell death as described in Section 2. Data represent the mean ± standard error of three independent experiments. Note that the deletion of RS218-derived genomic island 21 (RDI 21) abolished* E. coli* K1-mediated cytotoxicity.

For BMEC cytotoxicity, all mutants tested except RDI 21 showed BMEC death at levels similar to the wild type* E. coli* K1 RS218. The wild type* E. coli* K1 RS218 lacking RDI 21 showed significantly reduced levels of BMEC cytotoxicity (*P* < 0.05) (Table 3). Among other virulence factors encoded by the genomic island, previously our studies have identified cytotoxic necrotizing factor-1 (CNF-1) as an important toxin encoded by this genomic island that is required for* E. coli *K1 invasion of BMEC* in vitro* and demonstrated its role in producing meningitis in newborn rats* in vivo *[15]. To determine whether abrogation of cytotoxicity in RDI 21 is due to* cnf1 *deletion, we used isogenic* cnf1* mutant tested in cytotoxicity assays. The results revealed that* Δcnf1* produced BMEC death at levels similar to the wild type* E. coli* K1 RS218 (Figure 2) suggesting that RDI 21 gene(s)/factors other than CNF-1 are involved in* E. coli* K1-mediated cytotoxicity of BMEC.

## 4. Discussion 

*Acanthamoeba *is a Trojan horse of the microbial world. It acts as reservoir for many bacterial pathogens such as* Legionella pneumophila, Pseudomonas aeruginosa, Coxiella burnetii, Vibrio cholera,* and many others [19]. These bacterial pathogens survive and multiply inside* Acanthamoeba* under harsh environmental conditions and are transmitted to susceptible hosts. For instance, residing within amoeba has been suggested as the “training ground” during evolution of bacteria to become human pathogens [10, 20]. The evolution of one species to house inside another is a remarkable adaptation and consistent with the fundamental principle of natural selection to favour cooperation. In a landmark observation of Rowbotham [21], it was shown that* Legionella pneumophila* can survive intracellular of* Acanthamoeba*. The ability of* L. pneumophila* to resist grazing by* Acanthamoeba* suggested their long coevolutionary history combined with a series of adjustments ensuring bacterial survival and that grazing resistance may have influenced evolutionary gain of bacterial pathogenicity [20, 22]. Thus it is important to study interactions of bacteria that are human pathogens with the environmental* Acanthamoeba* to identify potential virulence determinants. Previous studies have shown that RDIs 12 (*sia *operon; prophage genes) and 22 (invasins (IbeA); toxins (alpha-hemolysin), metabolism) are crucial in* E. coli* K1 RS218 association with BMEC [12]. In contrast, our findings revealed that deletion of neither RDI 12 nor 22 had any effect on the wild type* E. coli* K1 association with* A. castellanii* keratitis isolate belonging to the T4 genotype. A likely explanation may be that* E. coli* K1 association with a nonphagocytic BMEC is driven by bacterial determinants. Thus the deletion of the genomic island containing adhesins would result in reduced association as observed by Xie et al., 2006 [12]. However, phagocytic nature of* Acanthamoeba* would result in bacterial uptake mediated by host amoeba, in addition to the presence of specific bacterial adhesins. Notably, when tested for their association with BMEC, both RDIs 12 and 22 showed significantly reduced levels of BMEC association which is consistent with previous findings of Xie et al. [12]. The ability of* E. coli* to survive within* A. castellanii* trophozoite stage as well as withstand transformation of the trophozoite form into the cyst form is a remarkable property. We believe that the genomic islands required for this process are of particular interest in our search for potential* E. coli* K1 virulence determinants. It was interesting to note that strains including plasmid cured RS218, the wild type* E. coli* K1 in which black hole was filled with K-12 HB101 DNA, RDIs 2 (prophage genes), 4 [adhesins (S fimbriae, antigen 43), protein secretion system (T5SS), iron uptake system], 9 (iron uptake system), 10 [toxins (peptide toxins)], 16 [protein secretion system (T2SS), K1 capsule biosynthesis], 18 (prophage genes), 21 [adhesins (P fimbriae, nonfimbrial adhesins), protein secretion system (T1SS)], and 22 [invasins (IbeA); toxins (alpha-hemolysin), metabolism] showed recovery from cysts at reduced levels compared with the wild type* E. coli* K1 RS218. Our previous findings revealed that type III secretion system (within the RDI 7) and* neuDB* genes cluster (within the RDI 16) is important for* E. coli* K1 interactions with* A. castellanii* [23, 24]. While being consistent with previous data, RDI 16 showed reduced recovery of* E. coli* K1 from cysts; however, there is a need to determine the role of RDI 7 in* E. coli* K1 interactions with* A. castellanii* as well as HBMEC and this will be addressed in future studies.

For BMEC, the results revealed that the deletion of RDIs 10 [toxins (peptide toxins)], 21 [adhesins (P fimbriae, nonfimbrial adhesins), protein secretion system (T1SS)], and 22 [invasins (IbeA); toxins (alpha-hemolysin), metabolism] reduced the ability of* E. coli* K1 to associate, invade, and survive BMEC. When comparing with the intracellular cyst survival assays, three RDIs, 10, 21, and 22, were important in their interactions with both BMEC as well as* A. castellanii*. Future studies are needed to precisely determine the role of specific virulence determinant(s) within each genomic island required for* E. coli* K1 interactions with* A. castellanii* and BMEC.

Consistent with previous findings [12], it was observed that RDI 12 deletion resulted in reduced* E. coli* K1 association with BMEC but had no effect on its invasion of BMEC. Similarly, it was observed that RDI 22 deletion resulted in reduced* E. coli* K1 invasion of BMEC but had no effect on its association of BMEC. In contrast, RDI 1 deletion affected neither association nor invasion of BMEC, while Xie et al. [12] showed reduced levels of invasion. This could be due to assay conditions such as 90 min [12] versus 60 min incubations in our assays. Given that assays are performed under static conditions in the absence of any immune factors, compared with the* in vivo* situation and under dynamic blood flow, it is believed that 60 min incubations provide ample opportunity for bacteria to interact with the host cells. Additionally, a reduced duration of incubation will limit the effects of extracellular factors that are shed during bacterial growth (e.g., LPS), as the generation time for* E. coli* under laboratory conditions is 15 to 20 min. Apart from this anomaly, the findings regarding* E. coli* K1 interactions HBMEC observed in this study are consistent with those observed by Xie et al. [12] and further suggest the need to identify virulence determinant(s) within these genomic islands.

In addition to meningitis,* E. coli* K1 sepsis is a serious complication with a mortality rate of >50%. It is the result of a high level of bacteremia and characterized by capillary leak, hypotension, and organ dysfunction [2]. Both lipopolysaccharide (LPS) [24] and non-LPS factors are involved in disease pathogenesis [20]. To identify potential determinants, various* E. coli* RS218-derived genomic islands (RDIs) deletion mutants of* E. coli* K1 were tested for their ability to produce BMEC death. The deletion of RDI 21 significantly reduced the level of BMEC death (*P* < 0.05) suggesting the presence of putative virulence determinants in this genomic island. As CNF-1 has been identified as an important toxin encoded by this genomic island that is required for* E. coli *K1 meningitis [15], it was tempting to test its role in BMEC death. Notably, Δ*cnf1* exhibited BMEC death at levels similar to the wild type* E. coli* K1 RS218 suggesting that RDI 21 gene(s)/factors other than CNF-1 are involved in* E. coli* K1-mediated BMEC death. This is not surprising as distinct bacterial determinants are likely required for sepsis and the blood-brain barrier crossing leading to meningitis. Findings observed in this study may lead to future discovery of potential targets for therapy against sepsis as well as to the identification of determinants required for bacterial survival in the environmental phagocyte.

## Figures and Tables

**Table 1:** Summary of *E. coli* K1 RS218 derived islands (RDI's) and its genomic islands mutants tested in the present study.

*E. coli* K1	
Identity	Function and potential virulence factor
K1 (RS218)	Isolate from a meningitis patient.
Plasmid cured RS218	Plasmid-free strain
RS218 complemented with HB101 DNA	Strain, in which genomic deletion (black hole) observed in RS218 was filled with respective DNA from HB101
RDI 1	**Invasins **(IcmF and IcmH).
RDI 2	**Prophage genes.**
RDI 4	**Adhesins** (S fimbriae, antigen 43). **Protein secretion system** (T5SS for antigen 43). **Iron uptake system **(Iro system and *hmu* system).
RDI 9	**Iron uptake system **(Ybt system).
RDI 10	**Toxins **(peptide toxin).
RDI 12	**Other virulence factors** (*sia* operon). **Prophage genes.**
RDI 14	**Prophage genes.**
RDI 15	**Metabolism **(sugar metabolism).
RDI 16	**Protein secretion system** (T2SS). **Other virulence factors** (K1 capsule biosynthesis).
RDI 17	**Metabolism **(phosphor-sugar metabolism).
RDI 18	**Prophage genes.**
RDI 21	**Adhesins** (P fimbriae, F17-like fimbriae, non-fimbrial adhesins, Hek and hemagglutinin). **Protein secretion system** (T1SS for hemolysin). **Invasins **(CNF1). **Metabolism **(D-serine catabolism).
RDI 22	**Invasins **(IbeA). **Toxins **(*α*-hemolysin). **Metabolism **(dihydroxyacetone, glycerol, and glyoxylate metabolism).

**Table 2:** RS218-derived islands (RDI's) mutants and their interactions with *Acanthamoeba castellanii*. The number of *E. coli* K1 c.f.u. interacting with *A. castellanii* was considered as 100% and the results of mutants strains are expressed as the relative change.

Identity	*A. castellanii *			
	Association	Invasion	Survival	Cyst survival
K1 (RS218)	100	100	100	100
Plasmid cured RS218	130.49 ± 25	26.19 ± 2*	75.19 ± 10*	25 ± 5*
RS218 in which black hole was filled with respective DNA from HB101	143.8 ± 19	32.74 ± 7*	147.84 ± 23	227.5 ± 23*
RDI 1	111 ± 12	96.42 ± 9	132.8 ± 14	93.75 ± 16
RDI 2	68.53 ± 7*	36.90 ± 3*	132.8 ± 11	63.75 ± 8*
RDI 4	246.53 ± 31	143.44 ± 20*	108.58 ± 15	55 ± 5*
RDI 9	52 ± 3*	76.19 ± 6*	65 ± 8*	10 ± 2*
RDI 10	45.9 ± 6*	51.78 ± 5*	63.66 ± 6*	8.75 ± 3*
RDI 12	83.62 ± 10	69 ± 11*	92.18 ± 10	158.75 ± 9
RDI 14	88.92 ± 15	81.54 ± 11	97.26 ± 9	165 ± 5
RDI 15	60.19 ± 8*	73.8 ± 12*	110.34 ± 18	136.25 ± 13
RDI 16	124.7 ± 18	116 ± 11	93.35 ± 7	60 ± 9*
RDI 17	49.95 ± 6*	83.92 ± 9	108.39 ± 16	172.5 ± 12*
RDI 18	117.97 ± 16	28.57 ± 2*	25.38 ± 3*	8.12 ± 1*
RDI 21	157 ± 14*	39.28 ± 4*	70.89 ± 6*	45 ± 3*
RDI 22	109.62 ± 9	38.69 ± 6*	9.76 ± 1*	16.25 ± 3*

(i) Association was calculated as follows: (number of bacterial c.f.u./number of amoebae) × 100 = bacterial association of *A. castellanii*.

(ii) Invasion was calculated as follows: (number of bacterial c.f.u./number of amoebae) × 100 = bacterial invasion of *A. castellanii*.

(iii) Survival was calculated as follows: (number of bacterial c.f.u./number of amoebae) × 100 = bacterial survival of *A. castellanii*.

(iv) Cyst survival was calculated as follows: (number of bacterial c.f.u./number of amoebae cysts) × 100 = bacterial survival of *A. castellanii* cysts.

*indicates a significant difference (*P* < 0.05 using paired *t*-test, one-tailed distribution) when data of mutant strains were compared with the wild type *E. coli* K1 data.

**Table 3:** RS218-derived islands (RDI's) mutants and their interactions with human brain microvascular endothelial cells (HBMEC). For associated, invasion, and survival assays, the number of *E. coli* K1 c.f.u. interacting with HBMEC was considered as 100% and the results of mutant strains are expressed as the relative change. For cytotoxicity assays, *E. coli* K1-mediated HBMEC death was considered as 100% and the results of mutant strains are expressed as the relative change.

*E. coli *	HBMEC			
	Association	Invasion	Survival	Cytotoxicity
K1 (RS218)	100	100	100	100
Plasmid cured RS218	27.41 ± 4.5*	92.52 ± 5.6*	70.86 ± 6*	104 ± 7.8
Black hole filled with HB101 DNA	31 ± 1.6*	62.86 ± 13*	163.5 ± 28*	87 ± 10
RDI 1	89.97 ± 1.2	112.8 ± 10.6	96.56 ± 8	97 ± 13
RDI 2	98.42 ± 2.6	72.5 ± 8.7*	91.6 ± 9.1	108 ± 14
RDI 4	93.6 ± 3.52	98.37 ± 0.8	96.76 ± 1.8	124 ± 2.3
RDI 9	101.47 ± 2.6	109.6 ± 4.4	106.5 ± 3.4	110 ± 12
RDI 10	61.12 ± 1.4*	58.5 ± 6.1*	55.47 ± 6*	92.8 ± 3
RDI 12	70.7 ± 8.3*	88.3 ± 7.1	107.5 ± 11	89.9 ± 9
RDI 14	105.84 ± 4.1	133.7 ± 22	96.7 ± 11	103 ± 4
RDI 15	53.97 ± 7.5*	115.9 ± 5.5	113.6 ± 3.1	118 ± 7
RDI 16	67.78 ± 3*	111 ± 13	156.1 ± 19*	127 ± 5.4
RDI 17	104 ± 1	69.77 ± 7.4*	60.4 ± 3.4*	105 ± 2
RDI 18	107.52 ± 1.6	159.2 ± 18*	108.6 ± 8.8	88 ± 1.8
RDI 21	32.82 ± 4.6*	25 ± 3.6*	17.95 ± 3*	14.5 ± 1.1*
RDI 22	77.9 ± 8.9*	61.53 ± 8*	50.45 ± 7*	127 ± 3.4

(i) Association was calculated as follows: (number of bacterial c.f.u. recovered/original inoculum) × 100 = bacterial association of HBMEC.

(ii) Invasion was calculated as follows: (number of bacterial c.f.u. recovered/original inoculum) × 100 = bacterial invasion of HBMEC.

(iii) Survival was calculated as follows: (number of bacterial c.f.u. recovered/original inoculum) × 100 = bacterial survival of HBMEC.

(iv) Cytotoxicity was calculated as follows: (sample value − negative control value)/(total LDH release − negative control value) × 100 = HBMEC cytoxicity.

*indicates a significant difference (*P* < 0.05 using paired *t*-test, one-tailed distribution) when data of mutant strains were compared with the wild type *E. coli* K1 data.
